# Relationship between polyunsaturated fatty acid composition in serum phospholipids, systemic low-grade inflammation, and glycemic control in patients with type 2 diabetes and atherosclerotic cardiovascular disease

**DOI:** 10.1186/s12933-018-0672-5

**Published:** 2018-02-16

**Authors:** Malgorzata Poreba, Pawel Rostoff, Aleksander Siniarski, Magdalena Mostowik, Renata Golebiowska-Wiatrak, Jadwiga Nessler, Anetta Undas, Grzegorz Gajos

**Affiliations:** 1 Department of Coronary Disease and Heart Failure, Faculty of Medicine, Jagiellonian University Medical College, The John Paul II Hospital, 80 Pradnicka Street, 31-202 Kraków, Poland; 2 Institute of Cardiology, Faculty of Medicine, Jagiellonian University Medical College, The John Paul II Hospital, Kraków, Poland

**Keywords:** Cardiovascular disease, Atherosclerosis, Type 2 diabetes, Glycemic control, Inflammation, Fatty acids

## Abstract

**Background:**

There are inconsistent data about the role of serum phospholipid fatty acid composition in patients with type 2 diabetes (T2DM) and atherosclerotic cardiovascular disease (ASCVD). The aim of the study was to investigate the relationship between serum phospholipid fatty acid composition, systemic low-grade inflammation, and glycemic control in high-risk T2DM patients.

**Methods:**

Seventy-four patients (26% women, mean age 65.6 ± 6.8 years) with T2DM (median diabetes duration 10 years) and documented ASCVD (74 with coronary artery disease, 26 with peripheral arterial disease) were enrolled in the study. Baseline HbA_1c_ was estimated using turbidimetric inhibition immunoassay. According to the median value of HbA_1c_ the patients were grouped into those with HbA_1c_ < 7.0% (< 53 mmol/mol) (n = 38) and those with HbA_1c_ ≥ 7.0% (≥ 53 mmol/mol) (n = 36). Serum phospholipid fatty acids were measured with gas chromatography.

**Results:**

Patients with HbA_1c_ ≥ 7.0%, compared with those with HbA_1c_ < 7.0% had similar composition of saturated and monounsaturated fatty acids in serum phospholipids, but had higher concentrations of linoleic acid (LA) and higher n-6/n-3 polyunsaturated fatty acid (PUFA) ratio as well as lower levels of eicosapentaenoic acid (EPA), total n-3 PUFAs, and the EPA/arachidonic acid ratio. We found that LA (r = 0.25; p = 0.03) and n-6/n-3 PUFA ratio (r = 0.28; p = 0.02) were positively correlated with HbA_1c_. Multivariate logistic regression analysis showed that n-6/n-3 PUFA ratio, hsCRP and T2DM duration were independent predictors of worse glycemic control in patients with T2DM and ASCVD.

**Conclusions:**

This study showed that glycemic control in high-risk T2DM patients with ASCVD was significantly associated with unfavorable serum phospholipid n-6/n-3 PUFA ratio and greater systemic inflammation.

## Background

Cardiovascular diseases are the main complications of type 2 diabetes mellitus (T2DM), accounting for approximately two-thirds of deaths in T2DM patients [1–4]. The underlying mechanisms linking T2DM with atherosclerotic cardiovascular disease (ASCVD) remain not fully understood. There is strong evidence that classic risk factors do not explain the higher risk of ASCVD in T2DM patients [5].

Multiple pathophysiologic processes may contribute to ASCVD in T2DM including hyperglycemia, hypoglycemia, insulin resistance or hyperinsulinemia, dyslipidemia, chronic low-grade inflammation, oxidative stress, endothelial dysfunction, vascular calcification, and hypercoagulability [4–11]. Numerous experimental and clinical studies have shown the close relationship between dysglycemia and increased risk for ASCVD, with an estimated 11–16% increase in cardiovascular events for every 1% increase in glycated hemoglobin (HbA_1c_) level [4]. It has been shown that HbA_1c_ levels of ≥ 7.0% (≥ 53 mmol/mol) were associated with unfavorable cardiovascular outcomes in T2DM patients with established atherosclerosis [12].

Epidemiological studies have demonstrated that serum fatty acid (FA) profile is an independent risk factor for ASCVD [5, 13]. Much evidence has been accumulated indicating that dietary or blood FA composition was significantly associated with impaired endothelial function, systemic inflammation, oxidative stress, β-cell dysfunction, and insulin resistance [14]. It has been also demonstrated that serum/plasma FA profiles are related to an increased risk of T2DM and its macrovascular complications [14].

Long-chain polyunsaturated fatty acids (PUFAs) and their derivatives can modulate many metabolic and inflammatory pathways in diabetic and nondiabetic subjects [15–19]. Epidemiological evidence has indicated that populations with high fish consumption had less risk of diabetes and ASCVD [20]. Although beneficial effects of PUFAs have been widely documented, the results of ORIGIN (Outcome Reduction with Initial Glargine Intervention) study, the world’s longest and largest randomized clinical trial in T2DM and prediabetes, showed that n-3 PUFA supplementation did not affect the risk of death from cardiovascular causes in T2DM patients [21]. Thus, the recently published science advisory from the American Heart Association does not recommend supplementation with n-3 PUFAs for individuals with T2DM to prevent coronary artery disease (CAD) [20]. On the other hand, the treatment with n-3 PUFA supplements seems to be reasonable for the secondary prevention of CAD deaths among patients with prior CAD [20].

There are limited and inconsistent data about the role of serum phospholipid FA composition in the pathophysiology of T2DM and diabetes-related ASCVD. In addition, very little is known regarding associations of dysglycemia with serum FA profiles and chronic inflammation in T2DM patients with documented ASCVD.

Therefore, the aim of the present study was to investigate the relationship between FA composition in serum phospholipids, systemic low-grade inflammation, and glycemic control in patients with T2DM and established ASCVD.

## Methods

### Patients

The study design and population sampling are described in detail elsewhere [22]. In brief, we assessed 126 following T2DM patients with a history of coronary artery disease (CAD) and/or peripheral artery disease (PAD). Exclusion criteria were the same as in our previous study [22]. Finally, 74 patients with T2DM and established CAD and/or PAD were enrolled. The median duration of diabetes was 10 (interquartile range [IQR], 6–15) years. Diabetic patients were grouped according to the median value of HbA_1c_ into those with HbA_1c_ < 7.0% (< 53 mmol/mol) (n = 38 individuals) and those with HbA_1c_ ≥ 7.0% (≥ 53 mmol/mol) (n = 36 patients).

This study was performed according to the Helsinki Declaration with the approval of the Ethics Committee of the Jagiellonian University Medical College (No: KBET/190/B/2012). Informed consent was obtained from all individual participants included in the study.

### Blood sampling and laboratory measurements

Fasting blood samples were obtained between 8 and 10 a.m. after overnight fast. Samples were processed 30–60 min after blood collection. Then serum samples were stored at − 70 °C until further analysis. Routine blood tests, such as complete blood count, lipid profile, serum creatinine were carried out by automated laboratory techniques. HbA_1c_ was estimated using turbidimetric inhibition immunoassay (TINIA).

Serum levels of saturated (lauric acid, C12:0; myristic acid, C14:0; palmitic acid, C16:0; stearic acid, C18:0; lignoceric acid C24:0) and unsaturated FAs: n-7 (palmitoleic acid, C16:1), n-9 (oleic acid, C18:1), n-3 (alpha-linolenic acid—ALA, C18:3; eicosapentaenoic acid—EPA, C20:5; docosahexaenoic acid—DHA, C22:6) and n-6 (linoleic acid—LA, C18:2; eicosadienoic acid C20:2; arachidonic acid—AA, C20:4) were measured with gas chromatography (Agilent Technologies 6890N Network GC Systems, Wilmington, De., USA). The detailed methods were described previously [22]. A concentration of serum FAs of phospholipids fraction was expressed in µmol/l.

High-sensitivity C-reactive protein (hsCRP) was measured by latex nephelometry (Dade Behring, Marburg, Germany). The serum levels of interleukin-6 (IL-6) and tumor necrosis factor α (TNFα) were evaluated by ELISA (R&D Systems, USA).

### Statistical analysis

Categorical variables were presented as numbers and percentages. Continuous variables were expressed as mean ± standard deviation (SD) or median and interquartile range (IQR). Differences between the groups were compared using the Student’s t test for normally distributed variables. The Mann–Whitney U test was used for non-normally distributed continuous variables. Data normality was verified by the Shapiro–Wilk test. Categorical variables were compared by the Fisher’s exact test or by the Pearson’s χ^2^ test, when appropriate. The Spearman’s rank correlation coefficient was calculated to measure monotonic trend between two variables. Stepwise logistic regression analysis was performed for determining the independent predictors of poor glycemic control in the study patients. The calibration and discrimination of the developed model were assessed using the Hosmer–Lemeshow statistic and the area under the receiver operating characteristic curve (AUC), respectively. Two-sided p values < 0.05 were considered statistically significant. All calculations were made using the STATISTICA version 12.0 PL software package (StatSoft, Inc., Tulsa, Oklahoma).

## Results

### Baseline characteristics

The baseline characteristics of the study patients, including comorbidities and medications are shown in Table 1. Of the total population, 66.2, 97.3, 67.6%, were obese, hypertensive, and dyslipidemic, respectively. Mean age of the study population was 65.6 ± 6.8 years. There were no sex differences in demographic and clinical characteristics.

**Table 1:** Baseline characteristics of the study population (n = 74)

Variable	n = 74	HbA_1c_ < 7.0% n = 38	HbA_1c_ ≥ 7.0% n = 36	p value
Age (years)	65.6 ± 6.8	66.0 ± 6.7	65.2 ± 7.1	0.62
Female gender, n (%)	26 (35.1)	10 (26.3)	16 (44.4)	0.10
Hypertension, n (%)	72 (97.3)	37 (97.4)	35 (97.2)	0.97
Hyperlipidemia, n (%)	50 (67.6)	24 (63.2)	26 (72.2)	0.41
Metabolic syndrome, n (%)	74 (100.0)	38 (100.0)	36 (100.0)	1.00
Obesity, n (%)	49 (66.2)	26 (68.4)	23 (63.9)	0.68
Waist circumference (cm)	106.5 ± 9.4	106.7 ± 9.1	106.2 ± 9.8	0.83
Body mass index, kg/m^2^	31.2 ± 3.6	31.1 ± 3.0	31.3 ± 4.1	0.81
Body fat (%)	34.1 ± 8.6	32.9 ± 7.4	35.4 ± 9.6	0.23
Visceral fat (%)	16.0 ± 4.7	16.4 ± 5.0	15.5 ± 4.2	0.53
Total body water (%)	47.6 (44.2; 49.3)	48.6 (44.2; 49.3)	47.2 (44.0; 49.1)	0.45
Muscle mass (kg)	55.2 ± 10.4	56.1 ± 8.6	54.0 ± 12.7	0.51
Medical history				
T2DM duration (years)	10 (6; 15)	9 (4; 10)	10 (7; 20)	*0.02*
CAD, n (%)	74 (100.0)	38 (100.0)	36 (100.0)	1.00
PAD, n (%)	26 (35.1)	10 (26.3)	16 (44.4)	0.10
Previous MI, n (%)	28 (37.8)	17 (44.7)	11 (30.6)	0.21
Previous PCI, n (%)	47 (65.5)	25 (65.8)	22 (61.1)	0.68
Treatment				
ASA, n (%)	74 (100.0)	38 (100.0)	36 (100.0)	1.00
Clopidogrel, n (%)	33 (44.5)	16 (42.1)	17 (47.2)	0.66
Beta blocker, n (%)	61 (82.4)	32 (84.2)	29 (80.6)	0.68
ACE inhibitor or ARB, n (%)	67 (90.5)	33 (86.8)	34 (94.4)	0.26
Nitrate long acting, n (%)	11 (14.9)	5 (13.2)	6 (16.7)	0.67
Calcium antagonist, n (%)	32 (43.2)	15 (39.5)	17 (47.2)	0.50
Statin, n (%)	68 (91.9)	34 (89.5)	34 (94.4)	0.43
Fibrate, n (%)	1 (1.4)	0	1 (2.8)	0.30
Diuretic, n (%)	22 (29.7)	9 (23.7)	13 (36.1)	0.24
MRA, n (%)	9 (12.2)	4 (10.5)	5 (13.9)	0.66
Metformin, n (%)	49 (66.2)	27 (71.1)	22 (61.1)	0.37
Sulfonylurea, n (%)	31 (41.9)	19 (50.0)	12 (33.3)	0.15
Acarbose, n (%)	1 (1.4)	0	1 (2.8)	0.30
DPP-IV, n (%)	3 (4.1)	2 (5.3)	1 (2.8)	0.59
Insulin, n (%)	32 (43.2)	9 (23.7)	23 (63.9)	*0.0005*
PPI, n (%)	22 (29.7)	9 (23.7)	13 (36.1)	0.24

Data are given as number (percentage) for categorical variables and mean (± standard deviation) or median (IQR) for continuous variables

Italic values indicate significance of p value (p < 0.05)

*ACE* angiotensin-converting enzyme, *ARB* angiotensin II receptor blocker, *ASA* acetylsalicylic acid, *CAD* coronary artery disease, *MI* myocardial infarction, *MRA* mineralocorticoid receptor antagonist, *PAD* peripheral artery disease, *PCI* percutaneous coronary intervention, *PPI* proton pump inhibitor, *T2DM* type 2 diabetes mellitus

Patients with worse glycemic control at baseline (HbA_1c_ ≥ 7.0%) had significantly longer diabetes duration as compared to those with HbA_1c_ < 7.0%. There was a higher proportion of insulin users among subjects with HbA_1c_ ≥ 7.0% (Table 1).

There were no significant differences in laboratory characteristics between studied groups except for higher HbA_1c_ values and increased hsCRP levels in patients with worse glycemic control (Table 2). In addition, the individuals with HbA_1c_ ≥ 7.0% had lower baseline concentrations of C-peptide.

**Table 2:** Baseline laboratory investigations (n = 74)

Variable	n = 74	HbA_1c_ < 7.0% n = 38	HbA_1c_ ≥ 7.0% n = 36	p value
HbA_1c_ (%)	7.0 (6.6; 7.5)	6.6 (6.5; 6.8)	7.5 (7.2; 8.3)	*<* *0.0001*
Insulin (µIU/ml)	21.5 (14.6; 33.6)	19.1 (14.6; 27. 9)	24.4 (14.8; 38.5)	0.15
C-peptide (ng/ml)	3.25 ± 1.40	3.61 ± 1.47	2.87 ± 1.24	*0.02*
TC (mmol/l)	3.86 ± 0.91	3.69 ± 0.85	4.04 ± 0.95	0.10
LDL-C (mmol/l)	1.91 (1.53; 2.64)	1.825 (1.45; 2.51)	1.99 (1.63; 2.90)	0.22
HDL-C (mmol/l)	1.24 ± 0.38	1.28 ± 0.36	1.21 ± 0.40	0.75
Tg (mmol/l)	1.35 (1.12; 1.92)	1.375 (0.99; 1.91)	1.34 (1.14; 1.99)	0.57
Creatinine (µmol/l)	83.7 ± 22.0	82.9 ± 17.8	84.5 ± 25.9	0.77
eGFR (MDRD) (ml/min/1.73 m^2^)	78.3 (70.0; 90.0)	81.8 (70.0; 90.0)	78.0 (64.0; 90.0)	0.76
hsCRP (mg/l)	1.54 (0.73; 2.71)	1.33 (0.52; 2.43)	1.87 (0.85; 4.39)	*0.02*
IL-6 (pg/ml)	1.99 (1.55; 2.79)	1.88 (1.36; 2.28)	2.17 (1.64; 3.13)	0.09
TNFα (pg/ml)	1.48 (1.28; 1.76)	1.43 (1.24; 1.68)	1.52 (1.40; 1.79)	0.22

Data are given as number (percentage) for categorical variables and mean (± standard deviation) or median (IQR) for continuous variables

Italic values indicate significance of p value (p < 0.05)

*eGFR (MDRD)* estimated glomerular filtration rate calculated by the abbreviated MDRD equation, *HbA*_*1c*_ glycated hemoglobin, *HDL-C* high-density lipoproteins, *hsCRP* high-sensitivity C-reactive protein, *IL-6* interleukin-6, *LDL-C* low-density lipoproteins, *TC* total cholesterol, *Tg* triglycerides, *TNFα* tumor necrosis factor alpha

### Fatty acid composition in serum phospholipids

Saturated FAs were the largest fraction in serum phospholipids, followed by n-6 polyunsaturated, n-3 polyunsaturated, and monounsaturated. Among the single FAs, palmitic acid constituted the largest proportion with 31.2%, followed by AA (17.3%), LA (14.3%), stearic acid (13.6%), oleic acid (9.0%), and DHA (9.0%).

**Fig. 1:**
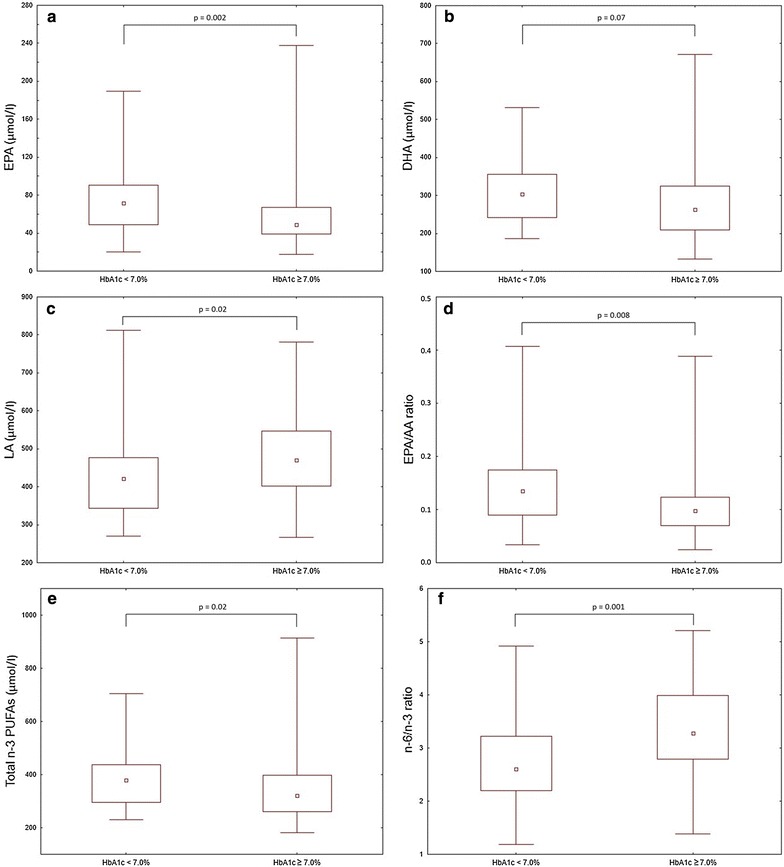
Eicosapentaenoic acid (EPA) concentration (**a**), docosahexaenoic acid (DHA) concentration (**b**), linoleic acid (LA) concentration (**c**), EPA to arachidonic acid (EPA/AA) ratio (**d**), total n-3 PUFAs concentration (**e**), and n-6 to n-3 PUFA ratio (**f**) in serum phospholipids of the study patients

There were no relevant intergroup differences in the composition of saturated and monounsaturated FAs in serum phospholipids (Table 3). T2DM patients with worse glycaemic control had significantly higher concentrations of LA and higher n-6/n-3 ratio as compared to diabetic individuals with HbA_1c_ < 7.0%. Furthermore, the study patients with HbA_1c_ ≥ 7.0% had lower levels of EPA, total n-3 PUFAs, and the EPA/AA ratio (Fig. 1).

**Table 3:** Serum phospholipid fatty acids composition in the study patients

Fatty acid	HbA_1c_ < 7.0% n = 38	HbA_1c_ ≥ 7.0% n = 36	p value
SFAs			
C12:0 (µmol/l)	1.73 (1.14; 2.84)	2.25 (1.39; 3.31)	0.17
C14:0 (µmol/l)	16.20 (15.09; 18.12)	18.31 (14.37; 20.92)	0.18
C16:0 (µmol/l)	940.62 (833.71; 1022.22)	973.60 (868.19; 1074.23)	0.50
C18:0 (µmol/l)	421.96 ± 92.87	439.14 ± 96.71	0.44
C24:0 (µmol/l)	24.17 (19.31; 28.40)	24.695 (20.38; 30.34)	0.99
MUFAs			
C16:1 (µmol/l	15.06 (12.33; 21.72)	14.56 (12.00; 19.79)	0.60
C18:1 (µmol/l)	270.77 (244.27; 332.28)	285.625 (246.25; 336.565)	0.75
n-3 PUFAs			
C18:3; ALA (µmol/l)	5.75 (4.73; 8.48)	7.21 (5.22; 8.94)	0.26
C20:5; EPA (µmol/l)	71.66 (48.65; 90.31)	48.55 (38.96; 66.86)	*0.002*
C22:6; DHA (µmol/l)	303.11 (241.62; 355.04)	263.25 (208.77; 325.18)	0.07
n-6 PUFAs			
C18:2; LA (µmol/l)	421.46 (343.43; 476.27)	471.02 (402.46; 546.30)	*0.02*
C20:2 (µmol/l)	16.39 (13.24; 18.33)	18.21 (14.39; 22.26)	0.14
C20:4; AA (µmol/l)	542.03 ± 95.38	561.92 ± 154.30	0.51
Total SFAs (µmol/l)	1439.66 ± 288.90	1431.66 ± 282.75	0.90
Total MUFAs (µmol/l)	286.15 (260.18; 356.12)	308.95 (261.43; 357.57)	0.77
Total n-3 PUFAs (µmol/l)	378.16 (296.06; 436.75)	320.70 (260.36; 396.61)	*0.02*
Total n-6 PUFAs (µmol/l)	986.24 ± 171.11	1065.54 ± 210.49	0.08
Total FAs (µmol/l)	3088.55 (2785.20; 3413.38)	3148.76 (2748.18; 3519.32)	0.65
n-6/n-3 ratio^a^	2.69 ± 0.78	3.32 ± 0.83	*0.001*
EPA/AA ratio	0.13 (0.07; 0.14)	0.10 (0.07; 0.12)	*0.008*
DHA/AA ratio	0.13 (0.09; 0.17)	0.50 (0.41; 0.59)	0.15

Data are given as number (percentage) for categorical variables and mean (± standard deviation) or median (IQR) for continuous variables

Italic values indicate significance of p value (p < 0.05)

*AA* arachidonic acid, *ALA* alpha-linolenic acid, *DHA* docosahexaenoic acid, *EPA* eicosapentaenoic acid, *FAs* fatty acids, *LA* linoleic acid, *MUFAs* monounsaturated fatty acids, *PUFAs* polyunsaturated fatty acids, *SFAs* saturated fatty acids

^a^The n-6/n-3 fatty acid ratio was calculated by measuring: linoleic, C18:2n-6; eicosadienoic, C20:2n-6; arachidonic, C20:4n-6 acids to estimate total n-6 PUFAs and alpha-linolenic, C18:3n-3; eicosapentaenoic, C20:5n-3; docosahexaenoic, C22:6n-3 acids to estimate the total n-3 PUFAs

### Correlations

We found that LA (r = 0.25; p = 0.03) and n-6/n-3 ratio (r = 0.28; p = 0.02) were positively correlated with HbA_1c_. No significant correlations were observed with regard to other FAs or ratios. In addition, we found a significant association between HbA_1c_ and hsCRP levels (r = 0.31; p = 0.008). There were no relevant associations between systemic inflammatory markers (hsCRP, IL-6 and TNFα) and serum phospholipid FA composition in T2DM patients with ASCVD.

### Univariate and multivariate logistic regression analyses

Statistically significant predictors of poor glycaemic control (HbA_1c_ ≥ 7.0%) in the study population are presented in Table 4. Multivariate logistic regression analysis demonstrated that n-6/n-3 ratio, hsCRP and T2DM duration were independent predictors of worse glycaemic control in our patients. The predictive model showed good cross-validated calibration and discrimination with Hosmer–Lemeshow χ^2^ = 10.73, p = 0.21 and AUC = 0.872, respectively.

**Table 4:** Univariate and multivariate logistic regression analysis of poor glycemic control (HbA_1c_ ≥ 7.0%) in the study patients

Variable	Univariate analysis		Multivariate analysis	
	OR (95% CI)	p value	OR (95% CI)	p value
Insulin therapy	5.70 (2.08–15.67)	< 0.001		
n-6/n-3 ratio	2.69 (1.41–5.14)	0.003	4.35 (1.72–10.96)	0.002
hsCRP (mg/l)	1.28 (1.01–1.62)	0.043	1.52 (1.04–2.21)	0.032
Total n-6 PUFAs (µmol/l)	1.25 (1.04–1.50)	0.020		
T2DM duration (years)	1.11 (1.02–1.21)	0.019	1.18 (1.04–1.32)	0.008
C18:2; LA (µmol/l)	1.01 (1.001–1.011)	0.037		
C20:5; EPA (µmol/l)	0.98 (0.97–1.00)	0.050		
Total n-3 PUFAs (µmol/l)	0.76 (0.63–0.93)	0.007		
C-peptide (ng/ml)	0.66 (0.45–0.96)	0.028		
EPA/AA ratio	0.001 (0.0001–0.51)	0.034		

*AA* arachidonic acid, *CI* confidence interval, *EPA* eicosapentaenoic acid, *HbA*_*1c*_ glycated hemoglobin, *hsCRP* high-sensitivity C-reactive protein, *LA* linoleic acid, *PUFAs* polyunsaturated fatty acids, *OR* odds ratio, *T2DM* type 2 diabetes mellitus

## Discussion

To our knowledge, this is the first study to show that a poor glycemic control (HbA_1c_ ≥ 7.0%) in high-risk diabetic subjects with ASCVD is associated with decreased levels of EPA, total n-3 PUFAs, and lower EPA/AA ratio in the serum phospholipid fraction. Furthermore, the patients with worse glycaemic control had increased serum concentrations of LA and hsCRP, as well as a higher n-6/n-3 ratio. In multivariate analysis, the n-6/n-3 ratio was the strongest predictor of poor glycaemic control, followed by serum hsCRP, and T2DM duration. Interestingly, we did not found any relevant intergroup differences in the composition of saturated and monounsaturated FAs in serum phospholipids.

It is well known that FA composition of serum phospholipids reflects dietary FAs intake during the preceding weeks as well as endogenous FAs metabolism, including FA synthesis (de novo lipogenesis) and FA desaturation, elongation, retroconversion, and oxidation [23, 24]. Recently published data indicate that meal frequency may also affect the FA composition of serum phospholipids in patients with T2DM [24].

### PUFAs and type 2 diabetes mellitus

Experimental and clinical studies showed that serum FA composition is abnormal in T2DM patients [13]. A few studies have demonstrated that elevated levels of palmitic (16:0), palmitoleic (16:1n-7), and dihomo-γ-linolenic (20:3n-6) acids and decreased concentrations of LA in both serum phospholipids and cholesterol esters are related to insulin resistance, metabolic syndrome and T2DM [23, 25]. Leeson et al. reported that higher levels of DHA in erythrocyte phospholipids were associated with improved endothelial function, particularly in young men who had some of the features of insulin resistance [15]. It has been also demonstrated that higher n-3 PUFA concentrations in red cell phospholipids were related to increased insulin sensitivity and a more favorable metabolic profile in middle-aged overweight men [26]. Takashi et al. found that T2DM patients with a history of prior myocardial infarction had significantly lower serum levels of EPA and DHA, as well as the EPA/AA and DHA/AA ratios as compared to diabetic patients without a history of myocardial infarction [16].

Over the past few decades, numerous investigations provided evidence for beneficial, cardioprotective effects of PUFAs, in particular of the n-3 family [13, 14, 17]. PUFAs, similarly to monounsaturated FAs, may decrease oxidative stress, inflammation and endothelial dysfunction, influence both insulin secretion and insulin resistance, and reduce diabetes risk [14]. In addition, n-3 PUFAs may slow down the progression of pancreatic β-cell dysfunction [27]. Several epidemiological studies showed that higher serum n-3 PUFA levels may be associated with the lower risk of T2DM [14, 20, 28, 29]. However, data from clinical studies have been conflicting.

It has been demonstrated that n-3 PUFA supplementation improved insulin sensitivity in Asian population of T2DM patients [30]. No benefits were found in Western populations [30, 31]. This finding suggests that the favorable effect of n-3 PUFAs on insulin sensitivity may be affected by ethnicity [31]. On the other hand, Kusunoki et al. found no significant association between HOMA-IR, which was used as a marker of glycemic control, and levels of EPA, DHA, and LA in serum phospholipids of Japanese patients with T2DM [32]. However, the authors did not evaluate the relationship between HOMA-IR and total levels of n-3 and n-6 PUFAs as well as the n-6/n-3 ratio [32]. Sawada et al. reported that 6-month EPA supplementation had no effect on plasma glucose, HbA1c, and HOMA-R in newly-diagnosed impaired glucose metabolism patients with CAD [33]. Interestingly, EPA treatment improved postprandial hyperglycemia, insulin secretion ability and hypertriglyceridemia, that might have beneficial effects on endothelial function and oxidative stress [33]. In our last randomized clinical trial, we demonstrated that treatment with 2.0 g of EPA-DHA per day for 3 months did not improve glycemic control in high-risk diabetic subjects [22]. We suggested that longer time of administration and/or higher doses of EPA-DHA as well as improvement in oral bioavailability of n-3 PUFAs may ameliorate glycemic control in T2DM patients.

The present study showed that high-risk T2DM patients with intensive glycemic control and HbA_1c_ level < 7.0% had higher levels of EPA and total n-3 PUFAs, and lower n-6/n-3 ratio, as compared to subjects with poor glycemic control. We hypothesize that the ratio of serum phospholipid n-6 to n-3 PUFAs may play a crucial role in control of glucose metabolism in high-risk T2DM patients. The clinical importance of n-6/n-3 ratio is still poorly understood. A few studies have demonstrated that the dietary n-6/n-3 ratio of 1:1 is the most beneficial for metabolic health, whereas it is approximately 15–20:1 in the present Western diet [34, 35].

In our study only LA was positively correlated with HbA_1c_. The serum phospholipid LA levels were significantly higher in subjects with worse glycemic control. This finding may at first seem surprising since n-6 PUFAs generally are associated with beneficial health effect. Sartore et al. demonstrated that T2DM subjects have lower LA levels and higher concentrations of highly unsaturated FAs [13]. Moreover, insulin sensitivity increases when saturated FAs are replaced by n-6 PUFAs [14].

It has been also demonstrated that LA in serum phospholipids and cholesterol esters was inversely associated with visceral adipose tissue and trunk fat, both of which are well-known contributors to insulin resistance and metabolic disease [36]. There are evidence that higher levels of n-6 PUFAs in plasma phospholipids, in particular LA, are linked to a lower risk of T2DM [29, 37].

On the other hand, some epidemiological studies and animal models suggest that n-6 PUFAs may promote adiposity and influence metabolic processes in various tissues [38]. There are a number of ways by which n-6 PUFAs can adversely affect metabolic pathways: (1) promoting tissue AA accumulation and increased production of pro-inflammatory eicosanoids, (2) reduced conversion of ALA into EPA, (3) reduced production of anti-inflammatory eicosanoids from EPA and DHA [39]. It has been suggested that n-6 PUFAs incorporated into phospholipids may be more susceptible to oxidative stress compared to n-3 PUFAs [24]. In addition, the oxidized metabolites of LA (OXLAMs), including bioactive 9- and 13 hydroxy-octadecadienoic acid (9- and 13-HODE) and 9- and 13-oxo-octadecadienoic acid (9- and 13-oxoODE), exert several pro-inflammatory and pro-atherogenic properties [40]. Some studies showed that higher plasma phospholipid n-6 PUFAs were associated with increased arterial stiffness as measured by carotid-femoral pulse wave velocity (cfPWV) [41, 42]. There is accumulating evidence from randomized controlled trials that replacement of dietary saturated FAs by LA significantly lowers serum total cholesterol (mostly by reducing low-density lipoprotein cholesterol) but does not support the hypothesis that this translates to a lower risk of death from CAD or decreased all-cause mortality [40].

Furthermore, it has been reported that LA intake was inversely associated with leukocyte telomere length (LTL) in female participants of the Nurses’ Health Study [43]. As an indicator of oxidative stress, DNA damage and cellular senescence, LTL has been postulated as a biomarker of aging and age-related chronic diseases, including CAD and T2DM [43, 44].

In our study, elevated levels of LA in serum phospholipids of patients with poor glycemic control could be caused by higher dietary intake of LA/lower frequency of meals or by altered pathways of n-6 PUFA metabolism. Unfortunately, we did not conduct a dietary survey in our patients. However, all participants were consulted by a cardiovascular physician and each patient received the same diet recommendations consistent with the European Society of Cardiology guidelines. Therefore, it seems unlikely, that the study patients with worse glycemic control had a different diet, particularly rich in linoleic acid, what could possibly result in higher concentrations of linoleic acid in serum phospholipids. We believe that higher serum phospholipid linoleic acid levels in patients with poor glycemic control could result from altered n-6 PUFA metabolism. As the parent compound for the family of n-6 PUFAs, LA can be elongated and desaturated to other n-6 PUFAs, such as γ-linolenic acid and AA. It has been reported that in diabetes, the fatty acid elongase and desaturase (i.e. Δ5- and Δ6-desaturase) activities decrease significantly, which may result in altered levels of PUFAs among patients with metabolic syndrome and T2DM [13, 45]. The desaturase enzymes are also regulated and modulated by many dietary and hormonal factors, including insulin [13, 45]. However, exogenous insulin does not seem to significantly influence phospholipid FA composition and desaturase activities in patients with T2DM [13].

As previously reported, a high intake of LA may disturb the metabolism and distribution of n-3 PUFAs [38]. It is well known that both LA and ALA compete for the same active site of microsomal ∆6-desaturase, the rate-limiting enzyme in long-chain PUFA biosynthesis [27]. It is possible that higher serum phospholipid LA concentrations in patients with poor glycemic control contribute to decrease of ALA metabolism and reduced synthesis of EPA and DHA, resulting in increased n-6/n-3 ratio.

### PUFAs and systemic inflammation

Numerous epidemiological and clinical studies showed that the high intake of n-3 PUFAs is associated with decreased inflammation [14]. Low-grade systemic inflammation as measured by serum concentrations of CRP is related to insulin resistance and ASCVD [46]. However, a potential mediating role of low-grade inflammation in the association between FA composition and insulin resistance is still unclear [46].

It has been suggested that n-3 and n-6 PUFAs cause opposite effects on systemic inflammation [34]. The eicosanoids derived from n-3 PUFAs generally have anti-inflammatory properties, while n-6 PUFA-derived lipid mediators are considered pro-inflammatory [47, 48]. LA is a precursor of AA, which may be converted to the pro-inflammatory prostaglandin E_2_ (PGE_2_) and leukotriene B_4_ [18]. On the contrary, eicosanoids derived from the EPA can down-regulate the biosynthesis of PGE_2_ [18]. The anti-inflammatory effects of n-3 PUFAs are related, in large part, to competition between EPA and AA as substrates for cyclooxygenases and to prevention of the conversion of AA into potent pro-inflammatory eicosanoids [18]. Thus, a high n-6/n-3 ratio may promote inflammatory diseases including ASCVD [16, 18]. The EPA/AA and DHA/AA ratios may reflect cardiovascular inflammation in patients with ASCVD and remain established markers of cardiovascular events [16].

Furthermore, n-3 PUFAs can serve as alternative substrates to produce less potent mediators, including 3-series prostaglandins and thromboxanes, and 5-series leukotrienes [18]. Experimental studies showed that EPA and DHA are also substrates for production of pro-resolving lipid mediators such as resolvins, maresins and protectins [18]. There is accumulating evidence that these bioactive compounds can influence glucose and insulin homeostasis and directly exert cardioprotective actions in vivo [18].

Serum concentrations of CRP in Western populations have been positively associated with some saturated (e.g. stearic acid) and monounsaturated FAs (e.g. palmitoleic and oleic acids), but inversely associated with LA and n-3 PUFAs such as ALA and EPA [49]. Kaska et al. found that both total saturated and monounsaturated FAs are positively correlated with serum hsCRP, whereas both n-6 and n-3 PUFAs show inverse correlation with this marker in patients with HbA_1c_ levels < 7.0% [48].

In addition, Poudel-Tandukar et al. demonstrated that CRP levels in a Japanese working population increased with a decreasing proportion of ALA and an increasing proportion of palmitic acid in men, and with an increasing proportion of dihomo-γ-linolenic acid in both sexes [49]. Similarly, studies among European populations have also disclosed an inverse association between serum ALA and CRP concentrations [49].

In our study, however, levels of saturated, monounsaturated and polyunsaturated fatty acids in serum phospholipids were not significantly associated with systemic inflammatory markers.

### Limitations

Our study has several limitations. First, the cross-sectional nature of the study did not allow us to infer causality. Second, the dietary fat intake was not assessed precisely, as our participants were provided dietary advice regarding low-fat and low-carbohydrate foods and caloric values. Therefore, we cannot unambiguously exclude the influence of a diet on the observed results. Finally, the sample size was relatively small in the present study and a larger sample would have provided more robust findings.

## Conclusions

In conclusion, this study showed that glycemic control in high-risk T2DM patients with ASCVD was significantly associated with unfavorable serum phospholipid n-6/n-3 PUFA ratio and greater systemic inflammation. There was no relevant relationship between serum phospholipid FA composition and systemic inflammation. These results confirm the beneficial effect of long-chain n-3 PUFAs in T2DM and support current recommendations for regular fish consumption.

## Abbreviations

AA: arachidonic acid
ACE: angiotensin-converting enzyme
ALA: alpha-linolenic acid
ARB: angiotensin II receptor blocker
ASA: acetylsalicylic acid
ASCVD: atherosclerotic cardiovascular disease
CAD: coronary artery disease
CI: confidence interval
DHA: docosahexaenoic acid
EPA: eicosapentaenoic acid
FAs: fatty acids
HbA_1c_: glycated hemoglobin
HDL-C: high-density lipoproteins
hsCRP: high-sensitivity C-reactive protein
IL-6: interleukin-6
LA: linoleic acid
LDL-C: low-density lipoproteins
MI: myocardial infarction
MRA: mineralocorticoid receptor antagonist
MUFAs: monounsaturated fatty acids
OR: odds ratio
PAD: peripheral arterial disease
PCI: percutaneous coronary intervention
PGE_2_: prostaglandin E_2_
PPI: proton pump inhibitor
PUFAs: polyunsaturated fatty acids
SFAs: saturated fatty acids
TC: total cholesterol
Tg: triglycerides
TNFα: tumour necrosis factor α
T2DM: type 2 diabetes mellitus
